# Multiple pregnancies, hepatitis C, and risk for hepatocellular carcinoma in Egyptian women

**DOI:** 10.1186/1471-2407-14-893

**Published:** 2014-11-29

**Authors:** Sania Amr, Emily A Iarocci, Ghada R Nasr, Doa’a Saleh, Jan Blancato, Kirti Shetty, Christopher A Loffredo

**Affiliations:** Department of Epidemiology and Public Health, University of Maryland School of Medicine, 660 West Redwood Street, Baltimore, MD 21201 USA; Departments of Oncology, Surgery and Microbiology, Georgetown University, Washington, DC, USA; Department of Community Health, Cairo University, Cairo, Egypt

**Keywords:** Hepatocellular carcinoma, Hepatitis C, Epidemiology, Pregnancy, Women’s health

## Abstract

**Background:**

The reasons for the worldwide sex disparity in the incidence of hepatocellular carcinoma (HCC) remain elusive. We investigated the role of multiple pregnancies on the associations between viral hepatitis C (HCV) infection and HCC risk among Egyptian women.

**Methods:**

We used data collected from blood specimens and questionnaires administered to female HCC cases and controls in Cairo, Egypt, from 1999 through 2009. HCV infection was defined as being sero-positive for either anti-HCV antibodies or HCV-RNA. Using logistic regression models we calculated odds ratios (OR) and 95% confidence intervals (CI) to estimate the associations between being HCV positive and HCC risk, and how it is modified by the number of pregnancies, after adjustment for other factors, including hepatitis B status.

**Results:**

Among 132 confirmed female cases and 669 controls, the risk of HCV-related HCC increased with the number of pregnancies. Women infected with HCV had higher risk for HCC if they had more than five pregnancies, as compared to those who had five or fewer pregnancies (adjusted OR (95% CI): 2.33 (1.29-4.22)). The association of HCV infection with HCC risk was significantly greater among the former (21.42 (10.43-44.00)) than among the latter (6.57 (3.04-14.25)).

**Conclusion:**

Having multiple pregnancies increases the risk of HCV-related HCC among Egyptian women, raising questions about the roles of estrogens and other pregnancy-related hormones in modulating HCV infection and its progression to HCC.

## Background

Hepatocellular carcinoma (HCC) is increasing worldwide and particularly in Egypt [1, 2] where the prevalence of hepatitis C viral (HCV) infection, a well-established HCC risk factor [3, 4], is the highest in the world [5]. Chronic infection with hepatitis C can lead to liver inflammation, cirrhosis, and ultimately to hepatocarcinogenesis [6, 7]. Also known is that HCC disproportionately strikes more men than women, worldwide [1, 8]. In searching for the mechanisms underlying gender difference in cancer incidence, investigators have examined risk factors before and after menopause, role of steroid hormones and specifically estrogens, with the assumption that the latter has a potential protective role [9–14]. Some investigators documented increased risk of HCC with increased exposure to estrogen and/or increased duration of higher estrogen levels [12, 13], and others found an inverse association [9, 10, 14]. Associations between parity and liver cancer risk among women were also investigated. Among Taiwanese women, Fwu et al. found that the higher the parity was the lower was the incidence of HCC [15]; whereas Chen et al. reported a standardized mortality rate (SMR) of 1.18 (95% CI: 1.06–1.30) for liver cancer among Taiwanese women with at least five children as compared to those in general population [16]. When multiparous (≥ 4 children) Italian women were compared to nulliparous, the relative risk for liver cancer was found to be 3.3 [17]. Therefore, to date, the studies of liver cancer risk and reproductive hormones (estrogen and others present during pregnancy) have yielded conflicting results.

Using data from our case–control study of HCC in Egypt [18], we investigated the role of multiple pregnancies in the association between HCV infection and HCC risk.

## Methods

### Study population

Detailed methods of recruitment, case confirmation, consent, and interview for the parent study were previously published [18]. Briefly, consecutive patients with presumed diagnosis of HCC were recruited from the National Cancer Institute of Cairo University from 1999 through 2009. They were included in the study only if their liver malignancy was confirmed as primary by either 1) pathology or cytology evidence, 2) alpha-fetoprotein (AFP) levels > 1000 ng/ml, or 3) AFP levels > 300 ng/ml along with evidence of single liver mass from an ultrasound or CT scan. All other cases, including non-malignant liver tumors or metastatic lesions, were excluded. Controls were recruited from the Orthopedic Department of the nearby Kasr El Aini Medical Center of Cairo University, which receives patients from the same geographical areas as the cases. They were frequency-matched to cases by rural versus urban birthplace, gender, and 5-year age category. The institutional review boards at the National Cancer Institute of Cairo University, Kasr El Aini Faculty of Medicine, and Georgetown University approved the study protocol [18].

### Questionnaire and biological specimen

For the parent study, each participant granted consent via either written or witnessed oral agreement. The 30 minute Arabic-language questionnaire was pilot tested before being administered by trained research assistants in face-to-face interviews; in addition to questions pertaining to socio-demographic characteristics that included age, education level and place of birth, environmental exposures, and medical histories, women were asked about number of pregnancies and live born children. A specimen of whole blood was collected from each participant and tested for serological markers of HBV and HCV, as described below [18].

### Laboratory assays

The HCV antibody was measured using an enzyme-linked immunosorbant assay (ELISA) from Abbott Laboratories (Wiesbaden, Germany). For HCV RNA determination, a reverse transcription-polymerase chain reaction was completed according to the method of Abdel Hamid et al., using nested primers from the highly conserved 5′-untranslated region (5′-UTR) of the HCV genome [19]. HBV core antibody (HBcAb) was determined using the CORZYME competitive immunoassay (Abbott Laboratories, Wiesbaden, Germany), while HBV surface antigen (HBsAg) was assayed using the enzyme immunoassay Auszyme method (Abbott Laboratories, Wiesbaden, Germany). Any participant was considered HCV infected if she tested positive for either HCV RNA or anti-HCV antibodies; similarly, detection of anti-HBV surface antigen or anti-HBV core antibodies was considered as HBV infection.

### Statistical analysis

HCV infection, the main predictor, was used as dichotomous variable, and the number of pregnancies was used as a continuous variable, but also as dichotomous based on the median in the controls. Age was used as continuous but also was grouped in three categories (≤ 45, < 45 to ≤ 55, and >55) for further descriptive analyses. Logistic regression models were used to calculate the odds ratio (OR) and 95% confidence interval (CI) to estimate the strength of the association between independent variables and HCC. Independent variables, including pregnancy number (continuous or categorical) were tested for their potential interactions with HCV infection. All models were adjusted for age, urban vs. rural birthplace, education (none versus some) and serological markers of HBV. All statistical analyses were performed using SAS, version 9.3.

## Results

A total of 132 female HCC cases and 669 controls participated in this study (with participation rates of 95% and 80%, respectively). Table 1 shows the characteristics of the cases and controls. Controls were significantly younger than cases and more likely to be born in urban areas. Approximately 50% of the controls and 70% of the cases were illiterate. A greater proportion of cases than controls reported more than five pregnancies and live births. The median number of pregnancies for cases and controls were 7 and 5, respectively. This difference between cases and controls was consistently noted in the agegroups (6 and 4 for the ≤ 45 y old group; 7 and 6 for the < 45 to ≤ 55 group; and 8 and 7 for those >55). Cases were more likely (81.1%) than controls (19.3%) to be HCV positive.

**Table 1:** **Characteristics and Infectious hepatitis statuses of women participants in the case–control study of hepatocellular carcinoma in Egypt**

	Controls N = 669	Cases N = 132	***p-value***
Age, mean (SD)	45.4 (14.9)	52.2 (10.5)	*<0.0001*
Age group N (%)			
≤ 45	332 (49.6)	32 (24.3)	
45 < to ≤55	165 (24.7)	46 (34.8)	*<0.0001*
> 55	172 (25.7	54 (40.9)	
Birthplace N (%)			
Urban	224 (33.6)	22 (16.8)	
Rural	443 (66.4)	109 (83.2)	*0.0001*
missing	2	1	
Education N (%)			
None	333 (49.8)	92 (69.7)	
Some	336 (50.2)	40 (30.3)	*<0.0001*
HCV infection N (%)			
No	540 (80.7)	25 (18.9)	
Yes	129 (19.3)	107 (81.1)	*<0.0001*
HBV infection, N (%)			
No	479 (71.6)	47 (35.6)	
Yes	190 (28.4)	85 (64.4)	*<0.0001*
Number of pregnancies,			
Mean ± SD	5.6 ± 3.3	7.3 ± 3.4	*<0.0001*
Median	5	7	
Categories, N (%)			
0- 5	374 (55.9)	39 (29.5)	
> 5	295 (44.1)	93 (70.4)	*<0.0001*
Number of children,			
Mean ± SD	4.7 ± 2.6	6.3 ± 2.7	*<0.0001*
Median	5	6	
Categories, N (%)			
0- 5	456 (68.2)	54 (40.9)	
> 5	213 (31.8)	78 (59.1)	*<0.0001*

HCV infection was significantly associated with HCC (OR (95% CI): 13.50 (8.09-22.53) after adjustment for age, birthplace, education, and HBV infection (Table 2, model 1). Assessed separately, the number of pregnancies was positively associated with HCC risk after adjustment for the same covariates, whether we used the variable as continuous (1.08 (1.02-1.16), or as dichotomous (≤ 5 versus > 5 pregnancies based on the median among controls) (1.84 (1.17-2.89), Table 2, model 2). When both variables, HCV infection and number of pregnancy (dichotomous), and an interaction term (HCV infection*pregnancy number) were included with the adjustment covariates in the regression model (Table 2, model 3), we found the interaction term to be significant (p = 0.02). Table 2 , model 3, illustrates the adjusted ORs and 95% CI of having HCC for the different strata; in the presence of HCV infection, the risk of having HCC was greater among women who had more than five pregnancies ((21.42 (10.43-44.00)) than among those with five or fewer ((6.57 (3.04-14.25)). And among those infected with HCV, having more than five pregnancies increased the odds of developing HCC (2.33 (1.29-4.22)) as compared to those with five or fewer pregnancies. In the absence of HCV, pregnancy number was not associated with HCC risk.

**Table 2:** **Multivariable regression analyses of the association between hepatitis C viral infection (HCV), pregnancy number and hepatocellular carcinoma among Egyptian women**

Variable	Adjusted OR (95% CI)*
**Model 1**	
HCV infection	
No	Reference
Yes	13.50 (8.09-22.53)
**Model 2**	
Pregnancy number	
≤ 5 pregnancies	Reference
> 5 pregnancies	1.84 (1.17-2.89)
**Model 3**	
HCV infection among women with ≤ 5 pregnancies	
No	Reference
Yes	6.57 (3.04-14.25)
HCV infection among women with > 5 pregnancies	
No	Reference
Yes	21.42 (10.43-44.00)
Pregnancy number among women without HCV infection	
≤ 5 pregnancies	Reference
> 5 pregnancies	0.72 (0.30-1.73)
Pregnancy number among women with HCV infection	
≤ 5 pregnancies	Reference
> 5 pregnancies	2.33 (1.29-4.22)
Dichotomized Pregnancy number * HCV interaction	
*p-value*	*0.02*

*Adjusted odds ratio (95% confidence interval); All models were adjusted for age, birthplace, education, and HBV infection.

Model 1 included HCV infection as the main variable with the adjustment covariates.

Model 2 included dichotomized pregnancy number (≤ 5 versus > 5) as the main variable with the adjustment covariates.

Model 3 included both HCV infection and dichotomized pregnancy number, and the interaction term dichotomized Pregnancy number*HCV interaction, in addition to the adjustment covariates.

## Discussion

Among Egyptian women in this case–control study, we found that multiple pregnancies increased the HCV-related risk for HCC. Although several previous studies have shown increased risk with estrogen exposure, this is the first observation addressing the interaction between pregnancy and HCV infection and its subsequent impact on HCC risk.

In searching for an explanation to the consistent higher incidence of HCC in men as compared to women, investigators postulated a role for estrogens and its protective effects; to date, the results have been inconsistent; some found increased risk of HCC with increased exposure to estrogen and/or increased duration of higher estrogen levels [12, 13], while others documented an inverse association [9, 10, 14, 15]. Estrogen was reported to inhibit the secretion of inflammatory interleukin from hepatic Kupfer cells in male mice, and was therefore hypothesized to have a protective effect on hepatic cells [9]. Our data showing that multiple pregnancies, with their recurrent high levels of circulating estrogens, increased HCC risk among HCV infected women do not support this hypothesis. On the other hand, exacerbation of chronic hepatitis C after delivery was reported among pregnant women carrying the viruses [20]. One of the explanations for this flare up is the effective rebound of the immune system, which is known to be suppressed during pregnancy [21, 22] most likely by the placental secretion of human chorionic gonadotropin (hCG) [23]; and after delivery, there is reactivation of the inflammatory cell response [20, 24, 25]. Multiple pregnancies, and thus multiple deliveries, can lead to multiple flaring episodes of the clinical signs of chronic hepatitis C, which, in turn, can lead to liver cirrhosis and carcinogenesis among women; a concept that is supported by our present results. Complex interactions between gender, serum interleukin levels, estradiol level, and HCC risk have also been reported [11]. In addition, Increased risk of liver cancer with increased parity have been previously reported in different populations [16, 17]; and so were progesterone and hCG as immunosuppressors during pregnancy [21].

Our study population was large enough to investigate women’s HCC associations with HCV infection and pregnancy number after adjustment for HBV. In addition, hepatocellular carcinoma diagnosis was confirmed by stringent criteria. At the same time, some limitations should be considered. The present analysis used a dataset established for a different original aim, and therefore we lacked some additional and potentially relevant variables, such as age at menarche, age at menopause, and age at first pregnancy. Future studies should also consider the use of oral contraceptives and hormone replacement therapy by women (neither of which were queried in our survey).

## Conclusion

The present study provides evidence for frequent pregnancies, and thus possibly hormonal factors during pregnancy or after delivery, as modulators of the HCV-related risk for HCC among Egyptian women. Future studies should consider 1) tracking the levels of Alanine Transaminase (ALT) (marker of liver injury) following delivery, and 2) assessing the relationship between multiple pregnancies and cirrhotic stages, in women with HCV infection.

## Abbreviations

HBV: Hepatitis B virus
HCV: Hepatitis C virus
HCC: Hepatocellular carcinoma.
